# Influence of Hempseed Meal on Fresh Goat Meat Characteristics Stored in Vacuum Packaging

**DOI:** 10.3390/ani13162628

**Published:** 2023-08-15

**Authors:** Virginia E. Zorn, Terry D. Brandebourg, Mary K. Mullenix, Aeriel D. Belk, Khim B. Ale, Frank W. Abrahamsen, Nar K. Gurung, Jason T. Sawyer

**Affiliations:** 1 Department of Animal Sciences, Auburn University, Auburn, AL 36849, USA; vez0001@auburn.edu (V.E.Z.); tdb0006@auburn.edu (T.D.B.); mullemk@auburn.edu (M.K.M.);; 2 Department of Agricultural and Environmental Sciences, Tuskegee University, Tuskegee, AL 36088, USA; kale8743@tuskegee.edu (K.B.A.); fabrahamsen@tuskegee.edu (F.W.A.); ngurung@tuskegee.edu (N.K.G.)

**Keywords:** color, hempseed meal, microbiology, storage life, tenderness, vacuum packaging

## Abstract

**Simple Summary:**

Hempseed meal (HSM) is a by-product of hemp manufacturing that can be an excellent source of crude protein and fiber in animal diets. Before accepting HSM as a feed ingredient throughout the food animal industry, the Food and Drug Administration’s Center for Veterinary Medicine requires extensive evaluation and documentation of feed ingredients across a species. Hemp contains nine essential amino acids and a healthy balance of the omega-3 and omega-6 fatty acids. HSM may be a potential new source of feedstuff for animals; however, an understanding of the effects of HSM on meat characteristics is limited in the literature. The current study was conducted to determine whether HSM will influence goat meat quality, shelf stability, or objective tenderness. Including HSM (0, 10, 20, or 30% inclusion rate in the total diet, on an as-fed basis) as a by-product ingredient caused changes to the surface color, postmortem pH, and instrumental tenderness measurements. However, HSM did not cause a deleterious effect on carcass characteristics.

**Abstract:**

The objective of this study was to determine the influence of hempseed meal (HSM) on goat meat characteristics. Goats (N = 10/treatment) were allocated to a diet concentration (0, 10, 20, or 30%) of HSM, fed for 60 days, and harvested. Carcass measurements were collected after chilling, and subsequently fabricated into wholesale subprimals. From the subprimals of the shoulder and leg, steaks were cut 2.54 cm thick, vacuum packaged, and assigned to laboratory methods: cook yield, instrumental color, lipid oxidation, microbial spoilage, and instrumental tenderness. HSM did not alter (*p* > 0.05) carcass characteristics, microbial spoilage, cook loss, or the thiobarbituric acid reactive substance (TBARS). However, a decrease in objective tenderness measurements (*p* < 0.05) was observed with greater concentrations of HSM supplementation in the diet. Instrumental surface color values for lightness (L*) indicated that steaks became lighter and less red (a*) as storage time increased (*p* < 0.05). Results suggest that HSM and storage time do not alter some goat meat traits, but HSM or storage time separately may influence goat meat quality. HSM may be an effective feed ingredient that does not alter carcass quality or meat yield.

## 1. Introduction

Hemp comes from the herbaceous species *Cannabis sativa* L. and is one of the oldest plants cultivated [1]. Throughout the hemp industry, plants have been cultivated to contain less than 1% of THC, unless grown in a stressful environment [2,3]. It has been reported that during hemp seed milling, roughly 90 to 99.6% of the oil within the hemp seed is extracted, resulting in a protein- and fiber-dense meal [3,4]. In the United States currently, hemp by-products are not approved for use as animal feeds. Unfortunately, there is a lack of research focused on the nutritional impact of feeding goats HSM, and there are even fewer studies referencing HSM and meat quality, shelf stability, or the textural influence of HSM on goat meat. Increasing the foundational knowledge of hemp for use in animal diets is important to expand scientific implications on meat characteristics and storage duration.

Healthier sources of protein that maintain the nutritional attributes of high quality meat in human diets have become a growing trend in today’s society [5]. There are many healthy options of meat proteins for consumers, but interest in goat meat has risen [6]. Globally, goat meat is considered a highly nutritious source of red meat [5,6,7]. Asian and African countries remain top producers of goat meat [7]. Goat meat is one of the most popular red meats in India and other regions, such as Southeast Asia and Africa, but it is often found unfavorable in Western countries [8,9]. Goat meat is often perceived as exhibiting a tough and stringy texture, and an unappealing color, in addition to a strong flavor and aroma [2,7]. Over the past decade, the demand for goat meat has increased due to an exponential growth in ethnic diversity within the United States [6,8]. In 2014, over 43 million pounds of goat meat were imported, and in 2020, it was reported that the United States imported roughly USD 945 million worth of sheep and goat meat [4,9]. 

It has been identified by consumers that the most important attribute of goat meat during the last few decades has been tenderness [9,10,11,12]. More specifically, previous research has indicated that goat meat can be less juicy and less tender, which can lead to consumers being discouraged from purchasing this source of protein [11,12]. Understanding the factors that are instrumental in affecting the quality of goat meat may aid in consumer acceptability of the product [5,13]. Studies have reported that a goat’s diet and physiological age may have lasting effects on the color stability and tenderness of goat meat [14]. Therefore, the objective of this study was to determine the influence of hempseed meal on fresh goat meat color, storage stability, and cooked qualities.

## 2. Materials and Methods

### 2.1. Raw Material

Spanish × Boer male goats (N = 40), 10 to 11 months in age, and with a mean body weight of 44.05 kg (±3.8 kg) were cared for and fed by Tuskegee University (Tuskegee, AL, USA) according to institutional animal care and use guidelines (PRN R07-2019-5). Goats were supplemented with varying rates of HSM (as-fed basis) as a percentage of the total diet (Table 1). Supplementation rates were 0 (control), 10, 20, or 30% of the total diets on an as-fed basis. The full diet and daily animal management during a 60-day feeding period is described previously [15]. After the 60-day feeding period, goats were transported to Auburn University for harvest. Goats were harvested at the Lambert-Powell Meats Laboratory using USDA humane slaughter standards under simulated commercial conditions. Carcasses were chilled after harvest procedures at 2.0 ± 1.0 °C for 24 h. Chilled carcasses were split between the 12th and 13th ribs, and the exposed loineye area was allowed to bloom for 15 min prior to collection of carcass measurements (backfat thickness, ribeye area, chilled carcass weight) by university trained personnel. The backfat of each carcass was measured using a stainless-steel ruler to the nearest cm. Ribeye areas were measured with a grid to the nearest cm^2^. Cooler shrink was calculated using the following formula: (Hot Carcass Weight − Chilled Carcass Weight) ÷ Hot Carcass Weight) × 100)). Carcasses were fabricated according to USDA institutional meat purchase specifications (roasting, barbeque, hotel, food service) for fresh goat meat [16]. Subprimals from the leg, loin, rack, and shoulder were weighed (PB3002-S, Mettler Toledo, Columbus, OH, USA) and recorded. After fabrication, subprimals were individually identified, and vacuum packaged (Cryovac Bone Guard Barrier Bag, Sealed Air, Charlotte, NC, USA), then stored in the absence of light for 7 days at 4 °C. At the conclusion of dark storage, subprimals were removed from their individual packaging and cut into 2.54 cm thick steaks using a BIRO bandsaw (Model 334, Biro Manufacturing Company, Marblehead, OH, USA). 

### 2.2. Packaging and Display Conditions

Steaks were vacuum packaged using a Reiser roll-stock form and film vacuum packaging machine (Optimus OL0924, Variovac, Zarrentin, Germany) in a forming film with an oxygen transmission rate of 0.4 cc/sq. m^2^/24 h/atm. Packages of steaks were individually identified and placed on the shelves of a multi-decked, lighted, refrigerated display case (Model TOM—label 60 DXB-N, Turbo Air Inc., Long Beach, CA, USA) for 21 days. Refrigerated cases operated at 3.0 ± 1.5 °C with four 45 min defrost periods every 24 h. Case temperatures were monitored using temperature data loggers (Model-TD2f, Thermoworks, American Fork, UT, USA). Steaks were distributed throughout each case and rotated daily to simulate shopping conditions. On days 0, 7, 14, and 21, steaks (*n* = 10/treatment/day) were removed for laboratory measurements. 

### 2.3. Instrumental Color

Instrumental color was measured using a HunterLab MiniScan EZ colorimeter, Model 45/0 LAV (Hunter Associates Laboratory Inc., Reston, WV, USA). Prior to the instrumental color readings, the colorimeter was standardized using manufacturer black and white tiles. Surface color values were obtained by reading through the unopened package using illuminant A, a 10° observer, and an aperture of 31.8 mm. The color value of each package was measured in three different locations to record the lightness (L*), redness (a*), and yellowness (b*). Lightness (L*) values are a measure of darkness to lightness, redness (a*) values reflect changes in the surface redness, and b* values are a measure of yellowness; larger values indicate a lighter, more red or more yellow surface color. Chroma is a measurement of total surface color used as a measure of brightness or dullness (a large value is a more vivid color) and was calculated using (√(b*)^2 + (a*)^2). The hue angle illustrates a shift in color from redness to yellowness (a large value is a greater shift in surface color from red to yellow) and was calculated using [tan^−1^ (b*/a)]. The Red:Brown (RTB) ratio was calculated using the equation (630 mm/580 mm) and a larger calculated value is indicative of a greater surface color change from red to brown. Relative values for deoxymyoglobin, metmyoglobin, and oxymyoglobin were determined using formulas provided by the AMSA Meat Color Measurement Guidelines [17]. Deoxymyoglobin (DMb) was determined using the equation (%DMb = {2.375 × [1 − (A473 − (A730)/(A525 − (A730)]} × 100). Metmyoglobin (MMb) was calculated using the equation (%MMb = {1.395 − [(A572 − A730)/(A525 − A730)]} × 100). Oxymyoglobin (OMb) was calculated using the equation [%OMb = 100 − (%MMb − %DMb)]. 

### 2.4. Purge Loss and pH

Packaged steaks were weighed on an analytical balance (PB3002-S, Mettler Toledo, Columbus, OH, USA) and recorded. After recording the package weight, each steak was removed from their individual package, patted dry using a paper towel and weighed. Purge loss was calculated using the following formula: [(packaged weight − unpackaged weight) ÷ packaged weight × 100]. The muscle pH of each sample was measured in three random locations by inserting a metal-tipped pH probe (Model-HI99163, Hanna Instruments, Woonsocket, RI, USA) into the geometric center of the steak and values were averaged to obtain one reading for each steak. Prior to measuring pH, the probe was calibrated (pH 4.0 and 7.0) using a 2-point standard buffer (Thermo-Fisher Scientific, Chelmsford, MA, USA). 

### 2.5. Thiobarbituric Acid Reactive Substance (TBARS)

Following previously published guidelines [18], steaks were trimmed of excess fat, diced into duplicate 2 g samples and placed into plastic centrifuge tubes. An amount of 8 mL of phosphate buffer (50 mM, pH of 7.0 at 4 °C) containing 0.1% EDTA and 0.1% n-propyl gallate as well as 2 mL of trichloroacetic acid (Sigma-Aldrich, Saint Louis, MO, USA) were added to each tube. Samples were homogenized for approximately 45 s and filtered through Whatman No. 1 filter paper. Samples of the clear aliquot were transferred in duplicate by pipetting 2 mL into 10 mL borosilicate tubes. After pipette transfer of the supernatant, 2 mL of the 0.02 M 2-thiobarbituric acid reagent (Sigma-Aldrich, St. Louis, MO, USA) was added to each tube. Tubes were boiled for 20 min in a 37 °C water bath then placed into an ice bath for 15 min. Absorbance was measured at 533 nm using a spectrophotometer (Turner Model—SM110245, Barnstead International, Dubuque, IA, USA). Absorbance values were multiplied using a factor of 12.21 to obtain the TBAR values (mg malonaldehyde/kg of meat). 

### 2.6. Microbiology

Using standard methods outlined in the USDA’s Bacteriological Analytical Manual [19], duplicate 10 g samples were removed from each steak and placed in a 3M Sample Bag with Filter Sterile (3M Corp., St. Paul, MN, USA) with 50 mL of 3M Butterfield Buffer (3M Corp., St. Paul, MN, USA). Filter bags were agitated for 2 min, and the remaining solution was diluted using a five-time serial dilution system. Dilutions were plated on duplicate corresponding Petrifilm^®^ (3M Corp., St. Paul, MN, USA) aerobic plate count (APC) plates. Plates were incubated at 35 °C in an incubation chamber (Model IB-05G, Lab Companion, Yuseong-gu, Daejeon, Republic of Korea), and then petrifilms were counted after 48 h and recorded as colony-forming units per gram (CFU/g).

### 2.7. Warner-Bratzler Shear Force and Cook Loss

Prior to cooking, each steak was removed from their respective package, blotted dry with a paper towel, and weighed on an analytical balance (PB3002-S, Mettler Toledo, Columbus, OH, USA). Each steak was placed on an aluminum wire roasting rack inside a convection oven (Model-VC5ED, Vulcan, Baltimore, MD, USA) preheated to 177 °C. Steaks were cooked until the internal temperature reached 71 °C, removed from the oven and allowed to cool to room temperature. Upon each steak reaching room temperature, cooked steak weights were recorded. Cook loss was calculated using the following equation: [(raw weight − cooked weight) ÷ (raw weight) × 100]. Six cores were obtained parallel to the muscle fibers from each steak and sheared once using the Warner-Bratzler Shear Force (WBSF) attachment on a TA XTPlus texture analyzer (TA-XT-2i, Texture Technologies Corporation, Hamilton, MA, USA) at a crosshead speed of 200 mm/min and a load cell of 5 kg. Peak force in newtons (N) for each core was obtained and recorded.

### 2.8. Statistical Analysis

Data were analyzed with SAS (ver. 9.4, SAS Institute Inc. Cary using the GLIMMIX) procedures, with treatment and day as the fixed effects and replication as a random effect for surface color, pH, microbiology, lipid oxidation, WBSF, and cook loss. For carcass traits and subprimal weights, treatment served as the lone fixed effect. The least square means were computed, and the significant (*p* < 0.05) F-value were separated using the pairwise *t*-test (PDIFF option).

## 3. Results 

### 3.1. Carcass Data

Regardless of inclusion rate, backfat (*p* = 0.48), carcass weight (*p* = 0.47), cooler shrink (*p* = 0.78), and ribeye area (*p* = 0.85) did not change with dietary HSM (Table 2). Additionally, the weight of the primal shoulder (*p* = 0.37), rack (*p* = 0.25), loin (*p* = 0.53), and leg (*p* = 0.75) from the goat carcass did not differ with increasing HSM inclusion rates (Table 3). 

### 3.2. Instrumental Fresh Color

Objective surface color values were measured over the storage period of 21 days. There was no interaction (*p* > 0.05) between HSM and the day of display on packaged goat steaks. However, the main effects of simulated storage are presented in Table 4. The surface color of goat steaks became lighter (L*) as the duration of storage increased (*p* < 0.05). Redness values (a*) and RTB decreased (*p* < 0.05), whereas the hue angle increased (*p* < 0.05) indicative of the surface color of goat steaks becoming less red as the duration of storage increased (Table 4). Lastly, steaks became less vivid (C*) as storage time during the simulated display increased (*p* < 0.05), suggesting the surface color deteriorated during the storage period. 

In addition to the impact of the storage day, the HSM inclusion percentage altered the surface color of goat steaks (Table 5). Increasing concentrations of HSM resulted in a darker (L*) surface color (*p* < 0.05). Additionally, the redness (a*) and yellowness (b*) values decreased (*p* < 0.05) as the concentration of HSM increased (Table 5). As well, steaks became less (*p* < 0.05) vivid (C*) as the percentage of HSM increased. 

### 3.3. Purge Loss and pH

There were no differences in purge loss (data not reported) of packaged goat steaks regardless of HSM (*p* = 0.88) diet inclusion or storage duration (*p* = 0.67). Ultimate pH values differed (*p* < 0.001) throughout the storage period but were within normal muscle pH ranges that would not alter surface color (Table 6). In addition, pH values were greater (*p* < 0.05) with the increasing concentration of HSM in the diet (Table 7).

### 3.4. Cook Loss

There was no interactive (*p* = 0.3353) effect between the concentration of HSM and the day of the simulated display. Nevertheless, cook loss percentages increased (*p* < 0.001) as the duration of storage time increased (Table 6), whereas cook loss did not differ (*p* = 0.4339) regardless of HSM concentration (Table 7). As expected, with a greater storage duration, moisture losses within the meat can occur resulting in an increase in cooking loss. 

### 3.5. Lipid Oxidation (TBARS)

There was no interaction (*p* = 0.322) between HSM and the day of display for lipid oxidation values. The lipid oxidation of packaged goat steaks increased (*p* < 0.001) throughout the duration of the storage period (Table 6), but the HSM concentration did not alter (*p* = 0.2090) lipid oxidation (Table 7). 

### 3.6. Microbiology

There was no interaction (*p* = 0.27) between the concentration of HSM and the day of the simulated display in measurements of microbial growth. Results indicate that throughout the storage period, microbial spoilage increased (*p* < 0.001) with longer storage times (Table 6), whereas the inclusion rate of HSM (*p* = 0.23) did not alter microbial spoilage growth (Table 7). 

### 3.7. Warner-Bratzler Shear Force

There was no interaction (*p* = 0.53) between HSM and the storage period; however, the current results suggest that, independently, the storage duration (*p* < 0.001; Table 6) and HSM concentration (*p* < 0.001; Table 7) can alter objective tenderness values. As expected, objective tenderness values were greatest on day 0. Interestingly, goat steaks required the least amount of force for tenderness measurements on day 7. 

## 4. Discussion

### 4.1. Carcass Data

The current results are in agreement with previous studies evaluating by-products such as HSM or peanut skins within animal diets [20,21,22]. Additional studies evaluating carcass characteristics of cattle fed hempseed cakes reported no changes in carcass characteristics such as carcass weights, dressing percentage, ribeye area, or backfat thickness [22] with the use of hempseed cake. It has been suggested that differences in carcass characteristics when feeding HSM are due to a lack of essential amino acids such as lysine causing a reduction in animal growth [22]. It is feared that by-product feeding can alter meat quality, but when feeding peanut skins to lambs, there were no differences in the cold carcass weights of lamb carcasses [21]. Moreover, some alternative ingredients such as peanut skins can increase ribeye area of lambs when fed at 20% but cause deleterious effects if usage approaches 40% of the diet [23]. 

### 4.2. Instrumental Fresh Color

The surface color results of goat steaks in the current study tend to agree with the limited previous literature on the use of by-products in animal diets or when using vacuum packaging for storage [12,24,25,26]. In a similar study, it was concluded that L*, a*, and b* values increased in lamb meat stored over a 28-day simulated retail display period [27]. Studies have suggested an increase in L* values could be due to the changes in the meat structure via protein denaturation, which allows for greater light dispersion [26]. It has been reported that a shift in redness could be responsible for increasing values of yellowness (b*) in goat steaks [26]. However, studies have indicated that red to brown and chroma (C*) values tend to decline as storage time increases in beef steaks [26,28]. 

Additional research concluded that goats fed 75% supplemental peanut skins within the diet had greater yellowness values than goats fed 0 or 25% supplemental peanut skins [25]. It is reported that intramuscular fat was not altered regardless of inclusion rate for peanut skins in the goat diet [25]. Additional studies have concluded that feeding supplements, such as conserved forages with concentrates, alfalfa grazing or alfalfa grazing with concentrates, and conserved forages to lambs did not alter surface color values [29]. Nevertheless, the surface color of goat steaks in the current study deteriorated as the storage time and concentration of HSM in the diet increased. It is plausible that the changes in surface color of vacuum-packaged goat steaks are associated with the partial pressure of the package. Furthermore, longer storage periods can allow for a greater purge in vacuum packaging that contains water soluble forms of myoglobin, resulting in greater affinity for atmospheric gases. Unfortunately, these factors were not measured during the current study.

### 4.3. Purge Loss and pH

The postmortem pH values of goat steaks were an average of 5.91, which is slightly higher than normal postmortem pH meat samples, which can be 5.55 for red meat [30]. At the time of goat harvest, each carcass was rinsed with a USDA FSIS-approved organic acid (lactic acid 2%). Studies have concluded that organic acids in combination with vacuum packaging may contribute to a decline in postmortem muscle pH [31,32]. The current results are inconsistent with previous studies reporting pH values measured on goat rib meat, which remained the same throughout the duration of their study [24]. Previous research has suggested that the lack of change in pH when using various packaging methods during storage could be caused by a decrease in the enzymatic breakdown of meat proteins, which can often alter pH values [24]. However, another study observed that various diet supplementation of tannin-rich peanut skins influenced goat carcass and meat quality [25]. However, the peanut skin supplementation did not alter ultimate pH among dietary treatments of peanut skins when measuring pH between the 12th and 13th ribs [25], whereas another study reported the reason for greater pH values postmortem could be due to malnutrition and stress [33]. Fresh meat characteristics can be influenced by pH; however, in the current study, postmortem pH is not a driver for highlighting the meat changes that occurred. 

### 4.4. Cook Loss

The current results tend to agree with previous studies reporting that cooking loss is related to the meat’s water holding capacity and protein integrity [34]. Recent studies have concluded that cooking losses can be altered by cooking temperature or ultimate muscle pH [35,36]. Factors such as protein denaturation occurring during the postmortem conversion of muscle to meat can cause pH values to decline due to increasing concentrations of lactic acid [37]. Greater pH values can cause an increased water retention leading to greater cook losses [38]. Surprisingly, previous studies suggest that some dietary feed supplements do not alter the cook loss of meat products [25,39]. 

### 4.5. Lipid Oxidation (TBARS)

The increases in lipid oxidation values in the current study suggest that storage duration is a greater influencer than by-product feed ingredients. It is well documented throughout the literature that many factors such as diet, gender, and storage duration are influencers of fresh meat characteristics during refrigerated storage. 

The current results for lipid oxidation contradict previous studies. These studies have reported peanut skins fed to goats will alter lipid oxidation [25]. It is plausible that variations in lipid oxidation of the loin were possibly not affected due to the limitation of variation in the fatty acid compositions [25]. It has been well documented previously that lipid oxidation values will increase as the storage duration of fresh and cooked meat increases [23,38,39,40,41]. However, studies have suggested that lipid oxidation can be the limiting factor for consumer-perceived quality and acceptability within meat and meat products [42]. Previous studies have indicated that when lipid oxidation exceeds 2.0 mg/kg of malonaldehyde, the product could be considered unacceptable, but the use of vacuum packaging with less than 2% residual oxygen in the package may slow lipid deterioration [30]. 

### 4.6. Microbiology

Microbial counts reached a value of 5.7 CFU/g, which is well below the established spoilage threshold of 7 log CFU/g [43], suggesting our results throughout the storage period can be considered fresh. The current results are consistent with those reported in recent studies [23,43,44,45]. Microbial counts have been reported to increase throughout the storage of lamb steaks during the first 14 days but subsequently plateau during the last 14 days of a 28-day storage study [23]. Numerous factors may affect the storage life of a meat product, such as the packaging, storage temperature, presence of fat, salt, and nitrites, and overall pH [44]. Interestingly, the current results suggest that HSM does not hinder the microbial growth of packaged goat steaks during simulated storage conditions. 

### 4.7. Warner-Bratzler Shear Force

It is plausible that the differences occurring in tenderness could be a result of variability in the endpoint cooking temperature, thereby causing a reduction in cook loss and subsequent improvement in shear force. Shear force values often decrease as marbling and carcass fatness values increase [6,32]. Additionally, tenderness values can be dependent on factors such as the antemortem practices, postmortem conversion to meat, muscles that were sampled, and cookery method [46,47]. However, contrary to the current results, it has been reported that dietary treatments may affect cooked meat tenderness [46,47]. 

## 5. Conclusions

As emerging by-product feed supplements are being incorporated into livestock diets, it is increasingly important to understand how these impact carcass quality and meat shelf-life. In this study, we provided a brief illustration of the influence of HSM within the goat diet on resulting goat meat. Overall, we demonstrated that, while various inclusion levels of this supplement have no impact on carcass characteristics, cook loss, or microbial growth, its use did affect some measures of meat quality over the shelf-life. Surface color, postmortem pH, and tenderness all fluctuated with changing dietary levels of HSM inclusion. These results suggest that a 10% inclusion level generally produces the most favorable results for meat quality. It would be advantageous for future research to evaluate the overall influence of HSM on sensory taste profiles of retail cuts from the goat carcass to further elicit any detrimental impacts of HSM as a by-product feed ingredient. 

## Figures and Tables

**Table 1:** Experimental diet composition (as-fed basis).

Feedstuffs	Treatment			
	0%	10%	20%	30%
Hempseed meal	0.0	10.0	20.0	30.0
Cotton Seed Hulls	20.0	20.0	25.0	16.0
Alfalfa meal	30.0	25.0	20.0	23.2
Cracked Corn	31.6	30.9	27.2	27.3
Soybean Meal	15.9	10.6	4.3	0.0
Cane molasses	2.5	2.5	2.5	2.5
Premix *	1.0	1.0	1.0	1.0
Total (%)	100.0	100.0	100.0	100.0

* Premix is a commercially available goat mineral comprised of 15.3% calcium, 8% phosphorus, 50 ppm selenium, 4000 ppm zinc, and 300,000 IU/kg vitamin A.

**Table 2:** The effects of HSM supplementation on carcass characteristics of goat meats.

Treatment					
	0%	10%	20%	30%	SEM *
Backfat Thickness (cm)	0.06	0.04	0.03	0.04	0.013
Cold Carcass Weight (kg)	27.6	26.8	25.6	25.6	2.36
Cooler Shrink (%)	1.02	1.01	1.02	1.02	0.008
Ribeye Area (cm^2^)	33.03	32.19	34.12	33.09	0.234

Cooler shrink is calculated as follows: (Hot Carcass Weight − Chilled Carcass Weight) ÷ Hot Carcass Weight) × 100)). * SEM = standard error of the mean.

**Table 3:** The effects of HSM supplementation on subprimal weights of goat meats.

Treatment					
	0%	10%	20%	30%	SEM *
Shoulder (kg)	6.79	6.56	6.28	6.25	0.246
Rack (kg)	3.02	3.13	2.85	2.79	0.127
Loin (kg)	2.86	2.79	2.75	2.54	0.157
Leg (kg)	6.19	5.99	6.02	5.91	0.193

* SEM = standard error of the mean.

**Table 4:** The influence of storage day on instrumental surface color of goat steaks.

Day					
	0	7	14	21	SEM *
L*	40.72 ^c^	42.84 ^b^	44.77 ^a^	44.70 ^a^	0.360
a*	17.69 ^a^	16.64 ^b^	16.58 ^b^	16.78 ^b^	0.152
b*	7.08 ^ab^	7.61 ^b^	7.56 ^b^	8.00 ^a^	0.121
C*	19.35 ^a^	18.33 ^b^	18.24 ^b^	18.62 ^b^	0.177
Hue (°)	23.77 ^b^	24.45 ^b^	24.37 ^b^	25.43 ^a^	0.252
RTB	3.02 ^a^	2.69 ^b^	2.59 ^c^	2.64 ^bc^	0.025

^a–c^ Mean values within a row lacking common superscripts differ (*p* ≤ 0.05). * SEM, standard error of the mean.

**Table 5:** The influence of hempseed meal inclusion treatment on instrumental surface color of goat steaks.

Treatment					
	0%	10%	20%	30%	SEM *
L*	43.62 ^ab^	44.32 ^a^	42.71 ^bc^	42.38 ^c^	0.360
a*	17.28 ^a^	16.83 ^b^	16.87 ^ab^	16.70 ^b^	0.152
b*	8.05 ^a^	7.81 ^ab^	7.54 ^b^	7.57 ^b^	0.121
C*	19.10 ^a^	18.57 ^b^	18.50 ^b^	18.36 ^b^	0.177
Hue (°)	24.89 ^a^	24.84 ^a^	24.01 ^b^	24.28 ^ab^	0.251
RTB	2.75	2.68	2.76	2.75	0.025

^a–c^ Mean values within a row lacking common superscripts differ (*p* ≤ 0.05). * SEM, standard error of the mean.

**Table 6:** The influence of storage day on fresh goat meat characteristics.

Day					
	0	7	14	21	SEM *
pH	6.07 ^a^	5.82 ^c^	5.94 ^c^	5.80 ^b^	0.033
Cook loss (%)	24.54 ^c^	30.77 ^ab^	32.78 ^a^	29.98 ^b^	0.844
TBARS	2.73 ^c^	2.75 ^c^	2.80 ^b^	2.85 ^a^	0.013
Microbial	3.66 ^c^	5.21 ^b^	5.70 ^a^	5.27 ^b^	0.082
WBSF	28.98 ^a^	19.40 ^c^	26.16 ^ab^	24.49 ^b^	1.095

^a–c^ Mean values within a row lacking common superscripts differ (*p* ≤ 0.05). * SEM, standard error of the mean.

**Table 7:** The influence of feeding treatment on fresh goat meat characteristics.

Treatment					
	0%	10%	20%	30%	SEM *
pH	5.86 ^bc^	5.84 ^c^	5.94 ^ab^	6.00 ^a^	0.033
Cook loss (%)	29.43	29.57	30.54	28.55	0.844
TBARS	2.78	2.78	2.80	2.77	0.010
Microbial	4.89	4.86	5.07	5.02	0.082
WBSF	23.08 ^b^	28.85 ^a^	25.04 ^b^	22.06 ^b^	1.094

^a–c^ Mean values within a row lacking common superscripts differ (*p* ≤ 0.05). * SEM, standard error of the mean.

## Data Availability

Not applicable.
